# The Double Face of Mucin-Type O-Glycans in Lectin-Mediated Infection and Immunity

**DOI:** 10.3390/molecules23051151

**Published:** 2018-05-11

**Authors:** Vasily Morozov, Julia Borkowski, Franz-Georg Hanisch

**Affiliations:** 1 University Children’s Hospital, Mannheim, Heidelberg University, Theodor-Kutzer-Ufer 1-3, 68167 Mannheim, Germany; Vasily.Morozov@medma.uni-heidelberg.de (V.M.); Julia.Borkowski@medma.uni-heidelberg.de (J.B.); 2 Schaller Research Group at the University of Heidelberg and the DKFZ, 69120 Heidelberg, Germany; 3 Institute of Biochemistry II, Medical Faculty, University of Cologne, Joseph-Stelzmann-Str. 52, 50931 Köln, Germany

**Keywords:** human blood group antigens, lectins, adhesins, mucin, MUC6, trefoil factor family 2 (**TFF2**), norovirus, human milk oligosaccharides, fucoidan

## Abstract

Epithelial human blood group antigens (HBGAs) on O-glycans play roles in pathogen binding and the initiation of infection, while similar structures on secretory mucins exert protective functions. These double-faced features of O-glycans in infection and innate immunity are reviewed based on two instructive examples of bacterial and viral pathogens. *Helicobacter pylori* represents a class 1 carcinogen in the human stomach. By expressing blood group antigen-binding adhesin (**BabA**) and **LabA** adhesins that bind to Lewis-b and LacdiNAc, respectively, *H. pylori* colocalizes with the mucin MUC5AC in gastric surface epithelia, but not with MUC6, which is cosecreted with trefoil factor family 2 (**TFF2**) by deep gastric glands. Both components of the glandular secretome are concertedly up-regulated upon infection. While MUC6 expresses GlcNAc-capped glycans as natural antibiotics for *H. pylori* growth control, **TFF2** may function as a probiotic lectin. In viral infection human noroviruses of the GII genogroup interact with HBGAs via their major capsid protein, VP1. HBGAs on human milk oligosaccharides (HMOs) may exert protective functions by binding to the P2 domain pocket on the capsid. We discuss structural details of the P2 carbohydrate-binding pocket in interaction with blood group H/Lewis-b HMOs and fucoidan-derived oligofucoses as effective interactors for the most prevalent norovirus strains, GII.4 and GII.17.

## 1. Trefoil Family Factor 2 (TFF2), a Lectin with Probiotic Activity?

Most reviews dealing with *Helicobacter pylori* focus on the disease-promoting capacities of the bacterium after colonization of the gastric mucosa—that is, the induction of inflammatory processes that may ultimately lead to the development of ulcers and gastric cancer [1]. However, it should be emphasized that more than 50% of the world’s population is actually infected by the bacterium, and that the majority of infected individuals do not suffer from any health problems caused by *Helicobacter pylori* during their lifetime. On the contrary, recent evidence from Anne Müller’s group suggests that persistent infection with the gastric bacterial pathogen may be linked to protection from allergic, chronic inflammatory, and autoimmune diseases [2]. In developed countries, where the pathogen has been systematically eradicated through medical care measures (in the US, down to levels of as low as about 10%), the incidence of autoimmune diseases (e.g., asthma, allergies, and chronic inflammatory diseases) has dramatically increased, reaching epidemic proportions.

The provided health benefits for the host could be regarded as mere side effects of the pathogen’s adaptation. During the 30,000 years of coevolution with its human host, *Helicobacter pylori* has learned to adapt its antigenic profile through, for example, Lewis antigen expression to escape the host’s immune detection. On the other side, the human host has also developed adaptive responses to the bacterium, and a partially symbiotic coexistence with the potential pathogen could be supported by the concerted secretion of antibiotic and probiotic factors as components of a system for regulatory growth control.

In 2004, a Japanese group provided evidence that organ-specific glycans capped with α-1,4-GlcNAc inhibit *Helicobacter pylori* growth by blocking cholesterol α-glucosyltransferase, which synthesizes the essential cell wall glycolipid of the bacterium [3]. This natural antibiotic is structurally associated with mucins secreted by deep gastric glands. For this reason, *Helicobacter pylori* exclusively colonizes the surface epithelium, where MUC5AC is the prevalent, if not only, mucin found in the mucous layer. The epithelia of deeper glandular portions of the stomach are never colonized by the bacterium due to the secretion of MUC6, a mucin that specifically expresses O-glycans capped with α-1,4-GlcNAc [4]. Strikingly, the same gastric glands (cardiac glands, mucous neck cells, antral glands) cosecrete a small protein of the trefoil factor peptide family, the human trefoil family factor 2 (***h*TFF2**, UniProt Q03403, chromosome 21) [5]. TFF peptides have long been speculated to interact in some way with mucins, as they were often associated and copurified with them, and were thought to form integral elements in the organization of multimeric mucin networks. Evidence was recently provided that ***h*TFF2** is a Ca-independent, pH-resistant lectin that binds with high affinity to the aforementioned α-GlcNAc-capped O-glycans on MUC6 [6]. This striking feature of ***h*TFF2** suggests that it may block the growth inhibitory potential of antibiotic glycans upon binding to them, and could accordingly be regarded as a probiotic factor in *Helicobacter pylori* growth control. In line with these considerations, we have demonstrated that ***h*TFF2** is actually able to reverse the growth inhibitory effect of porcine stomach mucin (PSM), which expresses antibiotic glycans similar to human MUC6 [7].

The functional involvement of ***h*TFF2** in the protection of the human host can become evident from its identification as a gastric stem/progenitor cell marker during *Helicobacter* infection [8]. Further evidence for a link between trefoil factor peptides and *H. pylori* infection was revealed by demonstrating an aberrantly increased epithelial expression of both ***h*TFF2** and MUC6, the two proposed antagonistic players, in an *H. pylori*-infected gastric antrum, incisura, and body [9]. The cosecreted ***h*TFF2** has also been claimed to regulate mononuclear cell inflammatory responses in *H. pylori* infection [10]. Further evidence for a functional involvement of ***h*TFF2** in defense mechanisms came from the observation of an accelerated progression of gastritis to dysplasia in the pyloric antrum of **TFF2^-/-^** mice infected with *H. pylori* [11].

## 2. The Lectin Domain of *h*TFF2

**Figure 1:**
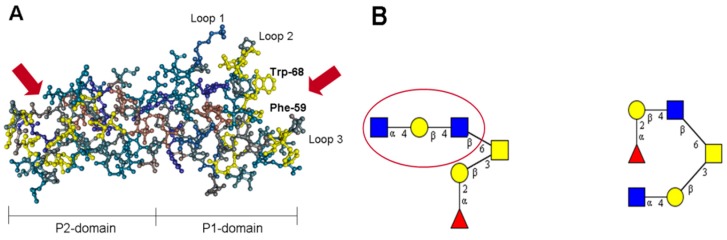
(**A**) Three-dimensional view of a porcine spasmolytic protein (***p*TFF2**). Ball-and-stick model of ***p*TFF2** with marked hydrophobic residues (yellow). For the P1 domain on the right side, loops 1–3 and amino acid residues Trp-68 and Phe-59 are highlighted. Red arrows point into the proposed binding pockets of the P1 and P2 domains. (**B**) Structural model of two hexasaccharides with antibiotic activity, which are O-linked to human MUC6 and porcine gastric mucin (both isomers). The symbols used for the designation of monosaccharides corresponds to the Consortium for Functional Glycomics nomenclature: fucose (red triangle), galactose (yellow circle), *N*-acetylglucosamine (blue square), *N*-acetylgalactosamine (yellow square).

Human trefoil factor family 2 (***h*TFF2**) is a secretory peptide with a molecular mass of 14,284 Da, that comprises 106 amino acid residues. In common with the two other members of the family, ***h*TFF1** and ***h*TFF3**, it is characterized by a specific pattern of cysteines that form disulfide bridges between Cys(I)–Cys(IV), Cys(II)–Cys(V), and Cys(III)–Cys(VI). ***h*TFF2** contains two TFF domains (P1 and P2), each representing 40-meric peptides with three conserved disulfide bridges that stabilize an extremely stable and rigid conformation (resistant to low pH and proteolysis), and form three characteristic loops. Gastric glycoforms of the TFF peptide are modified by N-linked glycans within the P1 domain, which terminate with monofucosylated *N,N′*-diacetyl-lactosediamine [12]. A unique feature of this small protein is the exposure of a hydrophobic patch lining a groove formed by loops 2 and 3 of the TFF domains (Figure 1A) [13]. This groove has been claimed to be responsible for protein–protein and protein–carbohydrate interactions [13,14]. Besides a series of hydrophobic amino acid residues, two sets of highly-conserved aromatic residues, Phe-59/Trp-68 in the P1 domain, and Phe-108/Trp-117 in the P2 domain, flank loop 3 (Figure 1A). According to the crystal structure of porcine **TFF2** (***p*TFF2**), the aromatic residues corresponding to Phe-59/Trp-68 in the P1 domain of ***h*TFF2** are located within strand 1 (Phe-59) and strand 2 (Trp-68) of the two-stranded anti-parallel β-sheet in the central core region of loop 3 [13]. These aromatic residues together with other aromatic/hydrophobic residues clustering around the β-turn in loop 2 (Gly-43, Phe-44, Gly-46, Ile-47, Phe-53, Val-63, Gly-65, Val-66) form a hydrophobic binding pocket that might be involved in the lectin-like activity of ***h*TFF2**. The glycotope defined by ***h*TFF2** corresponds to a trisaccharide with the structure GlcNAcα1–4Galβ1–4GlcNAcβ, which is part of a mucin-bound core 2 hexasaccharide [6] (Figure 1B). This trisaccharide was found to have a higher binding activity when compared to the corresponding disaccharide, GlcNAcα1–4Galβ , suggesting that a higher number of direct interactions with residues of the binding pocket is possible. 

## 3. Lectin-Based Host–Pathogen Interactions in *Helicobacter pylori* Colonization and Infection

Colonization of gastric mucosa by the gastric pathogen *Helicobacter pylori* (*H. pylori*) is a consequence of strong attachment of *Helicobacter* adhesins to cellular glycan structures on the host cell surface, particularly to the glycosylated gastric epithelial cell surface, and heavily glycosylated mucins. Bacterial persistence can result in chronic infection that may lead to stomach and duodenal ulcers, gastric cancer, or mucosa-associated lymphoid tissue (MALT) lymphoma [15], wherefore *H. pylori* was declared as a Class 1 carcinogen by the WHO (World Health Organization) in 1994. *Helicobacter pylori* harbors the cytotoxin-associated gene pathogenicity island (*cag*PAI), which encodes for the Cag bacterial type IV secretion system (T4SS) [16,17]. Via this secretion system, *Helicobacter* can interact with host cells and mediate the transfer of the oncogenic CagA protein. The presence of the *cag*PAI is clearly associated with an enhanced risk of developing gastric cancer [18]. CagA seems to have a dual role in directly priming pro-oncogenic signaling and indirectly leading to genomic instability, favoring neoplastic transformation [19].

*Helicobacter*’s attachment to the surface of mucous cells is directed by adhesins recognizing human blood group antigen (HBGA)-related carbohydrates expressed by epithelial cells of the gastric mucosa. The best studied lectins of the *Helicobacter* outer membrane protein family (HOPs) mediating adherence to host cells are the blood group antigen-binding adhesin (**BabA**) and the sialic acid-binding adhesin (**SabA**).

**Figure 2:**
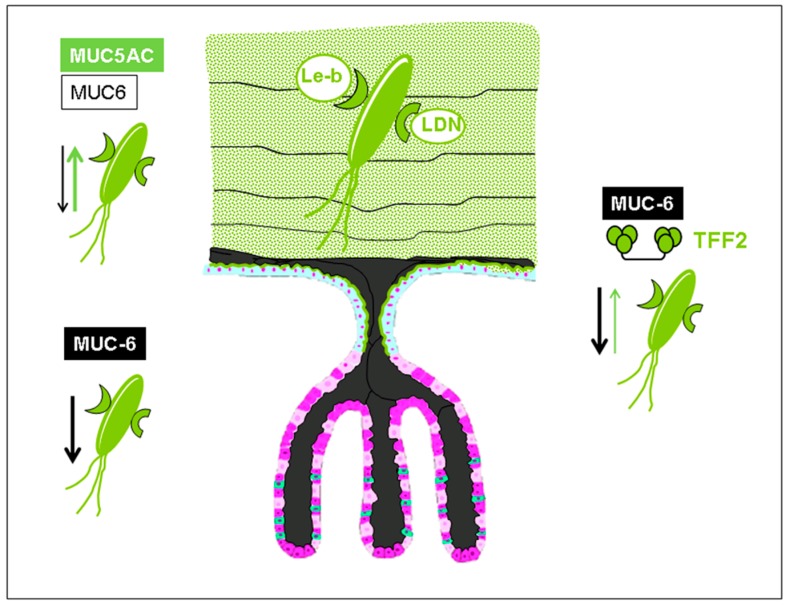
Schematic drawing showing *Helicobacter pylori* embedded in a mucin layer consisting mainly of MUC5AC that expresses Lewis-b and LacdiNAc (LDN) active O-glycans entrapping the bacterium via lectin interactions (blood group antigen-binding adhesin (**BabA**), LacdiNAc antigen binding adhesin **LabA**). In this way, the potential pathogen is kept away from getting into direct contact with the surface epithelium expressing the same blood group-related O-glycans. Deep gastric glands are producing MUC6, a mucin expressing antibiotic O-glycans that could be involved in growth control of the bacteria (refer to luminal portions of the glands, and to thin layers of MUC6 interscaled into the MUC5AC layer). Another control mechanism could be found in the interaction of antibiotic MUC6 glycans with a potentially probiotic cosecreted lectin, trefoil factor family 2 (**TFF2**).

**BabA** recognizes fucosylated type 1 glycans of the ABO and Lewis blood group (BG) systems present on glycolipids, glycoproteins, and mucins of the gastrointestinal tract [20]. Targeting only type 1 chain glycans underlines the tropism to foveolar epithelia of the upper stomach [21], thereby evading contact with antimicrobial mucin-covered type 2 chains of the deeper glandular stomach region (Figure 2) [3]. In detail, **BabA** detects the Fucα1–2 linkages (secretor fucose) to the type 1 chain Gal1–3GlcNAc core of the blood group O phenotype, representing the H1 antigen together with the corresponding Le^b^ antigen, where an additional branched fucose residue is added in an α1–4 linkage to the type 1 chain GlcNAc residue of the H1 antigen [20]. Additionally, **BabA** can use A and B blood group phenotypes (carrying terminal GalNAcα1–3 and Galα1–3 substitutions of the H1 antigen, respectively) and their corresponding Le^b^ antigens (ALe^b^ and BLe^b^) as receptors [22].

In contrast to **BabA**, the **SabA** adhesin preferentially attaches not only to sialyl-dimeric Lewis x (sdiLe^x^) carrying glycolipids and glycosphingolipids (GSLs), but also to the monomeric sLe^x^ and the related sLe^a^ form [23]. Sialylated glycoconjugates are rare in the healthy human stomach [24,25], but gastric inflammation and malignant transformation result in remodeling of the glycome [26], including a massive loss of neutral blood group ABH antigens and the expression of sialylated Lewis antigens [27,28]. In concert with this remodeling of the antigen repertoire of the gastric glycome, the pathogen adapts through preferential expression of the **SabA** adhesin [23]. Sialylated Lewis carbohydrate structures on the surface of leukocytes normally bind to selectins signaling injured tissue sites, thereby attenuating leukocyte circulation and favoring invasion into the injured tissue [29]. *Helicobacter* exploits the rendered mucous surface glycans as additional receptors for intimate adherence, performing selectin mimicry by binding the sialyl-(di)-Lewis x/a GSL [23].

Recently, another lectin of the HOP family, called **LabA**, was identified, targeting LacdiNAc (GalNAcβ1–4GlcNAc) residues. This lectin binds to a glycan motif, which in contrast to the targets of **BabA** and **SabA** is clearly restricted to the gastric mucosa. This may further explain the specific and restricted tropism of *H. pylori* to the gastric mucosa [30].

The HOP family also harbors adhesins other than the lectins **BabA**, **SabA**, and **LabA**, such as **OipA**, **HopZ,** and **AlpA/B** with diverse receptors [31], whose cooperation probably contributes to the chronicity and sequela of *Helicobacter* infection.

## 4. Adaption of the *Helicobacter* Lectin BabA to Host Glycome Polymorphisms

The majority of *Helicobacter* strains can bind to the antigens of several blood group and Lewis phenotypes (generalists), whereas some strains only attach to selected ones (specialists). These distinct blood group antigen preferences are solely determined by the **BabA** adhesin, and result from sequence and structural polymorphism in the *babA* gene [22,32]. Subtle differences in the amino acid sequence affect both Lewis binding strength and BG specificity [22]. The carbohydrate-binding domain (CBD) of **BabA** exhibits a high structural plasticity, which notably relies on the high sequence diversity in two (DL1 and DL2) of the three domains of the three-pronged binding site. The DL1 loop shapes the variable binding site for the terminal sugar moieties of the ABO antigen, thereby determining the adhesin’s blood group binding preference. The inward rotation of bulky amino acid residues (Asp, Asn, or Leu) at position 198, combined with a Pro at position 199, interferes with the protruding substitutions of the A (GalNAc) and B (Gal) phenotypes, leading to a selective binding of the H1 and Le^b^ antigens representing the O phenotype (specialist). However, the generalist **BabA** tolerates the more bulky sugar residues of the AB phenotype, enabling the binding of a variety of antigens (A, ALe^b^, BLe^b^, Le^b^, H1, and difucosylated GSLs, ALe^b^, BLe^b^, Le^b^). The amino acid triad Asp-Ser-Ser of the DL2 loop binds the reducing end of type 1 chain glycans, defining the specificity for type 1 chain glycans, where the Gal-GlcNAc linkage is β1–3 instead of β1–4, in contrast to type 2 chain glycans. The CL2 domain represents the anchor point of the binding site, being structurally conserved and wrapping around the secretor fucose. A disulfide bridge stabilizes a loop of nine conserved amino acids securing the α1–2 fucose binding [33]. Special phenotypes are also AB specialists, where the DL1 loop wraps more tightly around the terminal GalNAc/Gal moieties. In the inverse specialists, a mutation in the DL2 results in a lowered affinity, allowing a slight rotation of the DL2. Thus, despite exhibiting a bulky Pro-Leu sequence, it enables the binding of the protruding terminal GalNAc/Gal moieties, favoring the binding of the A/B, rather than the O, phenotype [32,33,34].

Despite the very high genetic variability of *H. pylori*, microevolution in individuals during chronic infection mostly does not affect host interaction factors such as **BabA**, since Le^b^-binding phenotypes remain quite stable during infection [35]. The considerable genetic variation in DL1 and DL2 results in adaptation to the host environment, which is due to positive selection [22], but adaptation refers rather to whole host populations. South American Indians dominantly express the blood group O phenotype, and **BabA** of those isolates preferentially recognizes H1, resembling the blood group O phenotype [22].

*Helicobacter* exhibits several mechanisms helping the bacterium to deal with the hostile gastric environment, which kills almost all other bacteria. To some extent, *Helicobacter* evades acidity by sensing when the pH gradient orientates to lower acid concentrations. It also neutralizes the pH in near proximity with the help of its urease activity, an enzyme which generates acid-neutralizing ammonia. Recently, Bugaytsova et al. additionally identified a pH-responsive reversible **BabA**-mediated adherence mechanism, which provided a profitable attachment–detachment system for its long-time colonizing and chronic lifestyle [36]. Binding of the **BabA** adhesin to Le^b^ was shown to be very sensitive to gastric acid, and dissociates from the receptor at a lower pH (a shift from 6 to 2). However, the binding can be reversed by a pH increase. Interestingly, strains from different intragastric regions exhibit differences in pH sensitivity. Variants from the upper, more acidic, corpus are more acid-resistant than those from the lower, less acidic, antrum, despite their original descent from the same ancestral strain [37]. These differences are due to amino acid alterations forced by pH-directed microevolution in particular, with variations in two domains proving to be responsible: the CBD, which affects Lewis-binding affinity, and the Velcro domain, which stabilizes binding through multivalent avidity effects. In detail, at a higher pH, the key-coil (aa199–202) in the CBD of **BabA** is clamped between a special salt bridge (pH sensor), thereby representing a better adapted binding site for Le^b^. At a lower pH, the salt bridge crashes, resulting in relaxation of the key-coil and distortion of the fucose-binding CL2 loop, leading to dissociation of **BabA** from Le^b^. The pH-sensitive variant, preferentially found in the antrum, exhibits an amino acid polymorphism at key position 199, favoring the already relaxed form, affecting Le^b^ binding. Helix 9 (H9), together with H1 and H10 outside of the CBD, forms the Velcro domain, harboring another critical position (aa428). Polymorphism at this position contributes to multimerization, and thus to acid sensitivity and avidity of Le^b^ binding. At a low pH, *Helicobacter* detaches from gastric epithelial cells and is released to the gut lumen, from where it subsequently moves back to the less acidic mucosal lining [36].

## 5. Mucin-Type O-Glycan Interactions in Viral Infections

Many viruses have also exploited lectins for their own benefits during infection. As an example, dengue viruses utilize a c-type lectin, dendritic cell-specific ICAM-3-grabbing non-integrins (DC-SIGN), on dendritic cells to enter the cells and replicate [38]. HIV-1 mainly employs DC-SIGN on dendritic cells to promote the transfer to CD4^+^ T lymphocytes and galectin-1 to facilitate attachment to CD4^+^ T cells [39,40]. Viruses themselves can encode lectins on their surface for cell targeting, among them noroviruses, rotaviruses, and influenza viruses. In the following section, we consider structural aspects of the norovirus-encoded lectins that define strain-dependent preferences toward HBGA glycan receptors.

## 6. Norovirus Capsid Proteins Exhibit Lectin-Like Functions

Noroviruses are non-enveloped single-stranded RNA viruses of the *Caliciviridae* family. Noroviruses are highly infectious, with transmission being mainly linked to person-to-person spread and contaminated food and water. The infection is usually self-limited and the symptoms generally last for ca. 48 h. However, the disease can be chronic or even life-threatening in elderly, immunocompromised people, and in children below the age of five years [41,42,43].

The norovirus virion is formed by an assembly of 180 copies of the major capsid protein (VP1) that dimerize to a T = 3 icosahedron core [44]. The minor capsid VP2 protein is also present; however, at a low copy number, its roles are not yet clearly defined [45]. The monomer of the VP1 major capsid protein consists of two domains, the shell (S) and protruding (P) domains, connected by a hinge. The S domain forms the core of the virion particles that surrounds and protects the RNA genome, while the P domain extends from the core, is involved in lectin-like receptor recognition, and defines strain diversity.

Based on variations within the VP1 gene, the norovirus genus is classified into seven genogroups (GI, GII, GIII, GIV, GV, GVI, and GVII) [46]. Noroviruses can infect mice (GV), cows (GIII), and cats (GIV). The GI, GII, and GIV noroviruses have been associated with gastroenteritis outbreaks in humans of all ages, with most infections caused by strains from the GII genogroup [47].

The norovirus major capsid VP1 is an example of the virus-encoded lectin. The murine VP1 capsid protein has been shown to interact with sialylated carbohydrates, while human norovirus VP1 proteins interact with polymorphic HBGAs through the P domain [48,49,50,51,52]. Although their precise role(s) is not known yet, the expression of HBGAs is generally accepted as a genetic factor that defines a person’s susceptibility to norovirus infection [53,54,55,56]. Human noroviruses interact with their HBGA partners in a strain-dependent manner, and these interactions seem to define their infectivity, spread, and impact on their evolutionary traits. The most prevalent genogroup II genotype 4 (GII.4) noroviruses seem to possess a broad and long-standing HBGA-binding profile [57], while the recently emerged GII.17 strain has gained higher HBGA-binding capabilities [58,59,60].

## 7. The P Domain of GII.4 Noroviruses

GII.4 noroviruses have been the predominant genetic cluster over the last decade, causing the vast majority of outbreaks, and six pandemics (US 96 [61]; Farmington Hills 2002 [62], Hunter 2004 [63], Den Haag 2006b [64], New Orleans 2009 [65]; and Sydney 2012 [66]). Interestingly, GII.4 noroviruses were circulating since the 1960s prior to their emergence in the 1990s. The factors involved in the emergence and the dominance of GII.4 noroviruses are unknown. However, a number of studies have suggested that rapid antigenic variations to produce a related strain allow GII.4 noroviruses to escape human herd immunity and cause reinfections [67,68]. In addition, the change in HBGA-binding sites has been suggested as another factor of the GII.4 evolution [69,70]. In recent years, numerous crystal structures of GII.4 norovirus P domains in the apo form and in complex with HBGAs have been determined, providing a basis for in-depth structural analysis [71,72,73].

**Figure 3:**
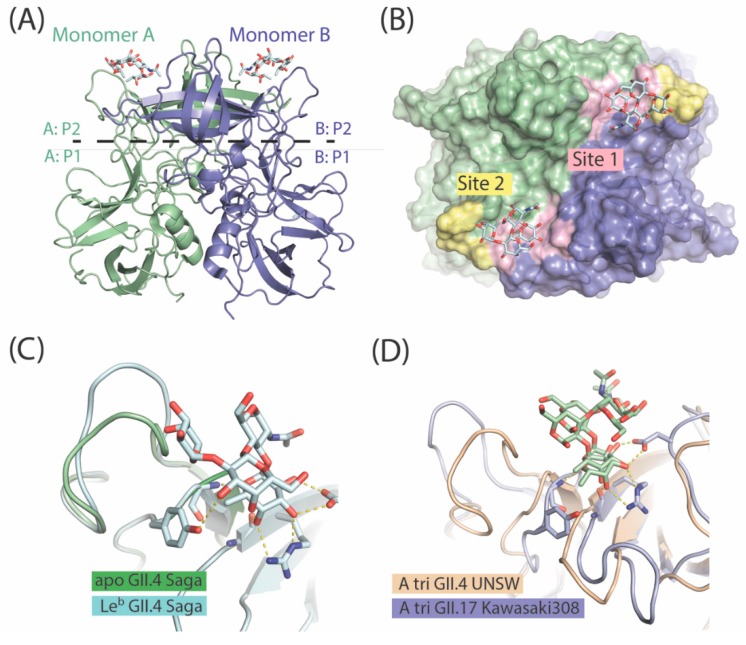
(**A**) Structure of the norovirus-encoded lectin—P (protruding) domain dimer. Each P domain monomer can be subdivided into P1 and P2 subdomains. P2 subdomains can recognize various types of human blood group antigens (HBGAs); (**B**) Site 1 and site 2 in the HBGA-binding pocket of GII noroviruses are displayed on the surface of the P domain dimer; (**C**) Superposition of the apo and Le^b^-tetra Saga-2006 GII.4 P2 subdomains; (**D**) Superposition of the A type trisaccharide GII.17 and A type trisaccharide GII.4 P2 subdomains.

The overall fold of the P domain is conserved among different GII.4 noroviruses. The P domain can be subdivided into two subdomains, P1 and P2. The P1 subdomain consists of seven β-sheets and one α-helix, while the P2 subdomain is a barrel-like structure with six antiparallel β-strands connected by surface-exposed small loops (Figure 3A). The P2 subdomain contains the HBGA-binding site located at the dimeric interface of the P2 domain dimer, and involves amino acid residues from both monomers (Figure 2B). Apparently, P domain dimerization is critical for HBGA recognition by GII noroviruses. In fact, an HBGA-local pH-sensitive destabilization of the P domain dimer is considered as the mechanism underlying the dissociation from salivary HBGAs, and the association to HBGAs on the GI tract epithelial surface [74].

Although the P2 subdomain represents the most solvent-exposed and hypervariable region of the capsid, residues that recognize the secretory fucose of HBGAs are highly conserved among various GII.4 strains, and contain Thr-344, Arg-345, Asp-374, Gly-443, and Tyr-444 (UNSW 2012 GII.4 numbering) amino acid residues. These residues are often referred to as site 1 in the HBGA-binding pocket (Figure 3B). Consequently, the binding of monofucosylated HBGAs (i.e., A and B trisaccharides) occurs in a remarkably similar manner in the early (1974) and more recent (1998, 2004, 2006, 2012) GII.4 noroviruses recognized [72,75].

A nearby loop (amino acids 390 to 395) surrounding the ABH-binding site can act as a secondary binding site (or supporting binding site) (Figure 3B). Interestingly, compared to the earlier strains, most of the GII.4 post-2002 strains contain a single amino acid insertion in this loop. This provides additional flexibility to the loop, allowing additional coordination contacts with longer saccharide chains and the binding of secretor difucosyl Lewis HBGAs [72,74]. As shown by X-ray crystallography studies in the Le^b^-hexa GII.4 (TCH05 2004), the Le^b^-tetra GII.4 (Farm 2004), and the Le^y^-tetra GII.4 (Saga 2006) (Figure 3C) norovirus P domains, the loops repositioned themselves to accommodate an additional Lewis fucose moiety.

The overall amino acid composition of the loop (the secondary binding site) is more diverse among strains. These variations can be involved in fine-tuning the specificity of GII.4 noroviruses toward specific Lewis HBGA types (Le^b^_,_ BLe^b^, Ale^b^) or precursor disaccharides (type 1 vs. type 2). Indeed, ELISA, SPR, and STD NMR in-solution experiments showed preferences of GII.4 VLPs (strains Ast6139 and Narito104) toward type 1 HBGAs [76,77]. Similar to our studies, the higher affinity of the pandemic GII.4 UNSW strain toward difucosylated Lewis HBGAs (Lewis b HBGAs) and H type 1 HBGAs has been confirmed in neoglycolipid-based arrays [78].

Lastly, preferences of GII.4 noroviruses toward specific HBGA types may not only be dictated by the structure of the P domain, but also by a proper orientation of the HBGA epitope on the surface of the target cell. Nasir et al. showed that, in the phospholipid membrane environment, GII.4 norovirus VLP binding to glycolipid-conjugated type 2 structures (ALe-y, Le-y, Le-x) was very limited due to the constrained accessibility of bulky VLPs to the (1,3)-linked fucose residues [79]. The same considerations may be relevant in solid-phase binding studies of VLPs on short carbohydrate chains in the size range of tri- to tetrasaccharides.

## 8. The P Domain of GII.17 Noroviruses

Since the winter period of 2014/2015, a novel GII.17 norovirus variant has been causing an increasing number of sporadic outbreaks, globally. In Asian countries, the emergent GII.17 strain has even replaced the previously dominant GII.4 genotype [58,59,80,81,82]. A possible explanation for this phenomenon is the absence of a herd immunity against GII.17 norovirus strains, due to the lack of previous exposure to rare GII.17 noroviruses and/or a large number of antigenic variations in novel GII.17 strains. In addition, faster VP1 evolution, due to more error-prone viral RNA-dependent RNA polymerase, can also contribute to the prevalence of GII.17 strains. Lastly, the change in the HBGA-binding profile to gain broad reactivity has been suggested as another factor in the spread of GII.17 strains.

Structural analysis of the rare Saitama T87 2002, and epidemic Kawasaki308 2014 and Kawasaki323 2015 GII.17 strains showed that the P domains of GII.17 strains are folded similarly to GII.4 and other human norovirus strains [83]. Most variations between GII.17 strains occurred within the HBGA-binding pocket or nearby, in the solvent-exposed loops. In the Saitama T87 2002 strain and other pre-epidemic GII.17 variants, the ABH fucose-binding site (site 1) was formed by the residues (Thr-348, Arg-349, Asp-378, Gly-443, and Val-444). In the epidemic GII.17 strains, Kawasaki308 and Kawasaki323, an amino acid mutation of Val-444 to Tyr-444 matched the ABH fucose site of the pandemic GII.4 noroviruses (Figure 3D). Considered in the context of multivalent glycan–virus interactions, this subtle mutation can in fact significantly impact the overall avidity of binding and increase fondness of novel GII.17 strains toward HBGAs. To support this hypothesis, site-directed studies have shown that a Y443A mutation can abolish the HBGA-binding capabilities of the GII.4 VA387 1996 norovirus [16].

Additionally, GII.17 strains are different from GII.4 noroviruses in the solvent-exposed loops that not only define antigenicity of the strains, but can also be involved in receptor binding [83]. Compared with the pandemic GII.4 strains, the secondary binding site in epidemic GII.17 noroviruses is positioned farther away [60] (Figure 3D). Its roles in HBGA coordination by the GII.17 noroviruses, if any at all, are not yet clear.

## 9. Specificity vs. Valency Aspects in Carbohydrate Recognition by Noroviral Lectins

Accumulating evidence suggests that fucose, as part of histo-blood group antigens or Lewis-like antigens, plays an essential role in the lectin-like recognition by the VP1 capsid protein of GII norovirus strains. This holds particularly true for the highly infectious GII.4 and the recently emerged GII.17 noroviruses. ABH and Lewis fucoses of blood group antigens are introduced by the FUT2 and FUT3 fucosyltransferases, and homozygous recessive individuals lacking these enzymes’ activities were less susceptible, or even resistant, to an infection by certain strains in human challenge and outbreak studies [54,70].

Based on the knowledge of carbohydrate specificities, a series of therapeutic measures are currently being evaluated. Besides vaccine-based approaches to combat the virus, one of the alternative strategies to prevent norovirus infections is based on food additives such as human milk oligosaccharides (HMOs). Human milk oligosaccharides represent an ideal source of potential competitors for viral glycan receptors, which mimic the structures of blood group-active mucin-type O-glycans. The trisaccharide 2’-fucosyllactose (2′FL) is able to block norovirus binding quite efficiently [60,84], and has gained market approval as a safe food additive. We could also provide evidence for other milk oligosaccharides in the high-mass range that exert even stronger competitive effects on norovirus binding to gastric mucins [78]. During these studies, we observed that the oligovalency of fucosyl residues in hepta- to decasaccharides provided increased promotion of competitive effects on norovirus binding. This became even more evident when L-fucose dendrimers with varying degrees of substitution, but no blood group relationship, were compared with respect to their competitive activities. High valency of α-L-fucose, with no structural relationship to blood group structures is also a feature of natural polysaccharides belonging to the group of polyfucoses or fucans. Among fucoidans, those of the brown algae have previously attracted much attention, as they were claimed to exert a series of health-beneficial effects [85]. In line with these findings, we were able to demonstrate a blood group-independent competitive anti-norovirus effect exerted by α-L-fucosyl residues of F-fucoidan and desulfated/fragmented processing products on viral capsid binding to gastrointestinal mucins.
